# Boosting the sterile insect technique with pyriproxyfen increases tsetse flies *Glossina palpalis gambiensis* sterilization in controlled conditions

**DOI:** 10.1038/s41598-020-66850-9

**Published:** 2020-06-19

**Authors:** L. Laroche, S. Ravel, T. Baldet, R. Lancelot, F. Chandre, M. Rossignol, V. Le Goff, M. Duhayon, J.-F. Fafet, A. G. Parker, J. Bouyer

**Affiliations:** 1 Intertryp, IRD, Cirad, Univ Montpellier, Montpellier, France; 2 ASTRE, Cirad, INRA, Univ Montpellier, Montpellier, France; 3 MIVEGEC, IRD, CNRS, Univ Montpellier, Montpellier, France; 4 3F Innovation, Saint-Amarin, France; 5 Insect Pest Control Laboratory, Joint FAO/IAEA Division of Nuclear Techniques in Food and Agriculture, IAEA Vienna, Austria; 6 Present Address: Roppersbergweg 15, 2381, Laab im Walde, Austria

**Keywords:** Model invertebrates, Assay systems

## Abstract

Tsetse flies (Diptera: *Glossinidae*) are the main vectors of animal and human trypanosomoses in Africa. The Sterile Insect Technique (SIT) has proven effective in controlling tsetse flies when applied to isolated populations but necessitates the production of large numbers of sterile males. A new approach, called boosted SIT, combining SIT with the contamination of wild females by sterile males coated with biocides has been proposed for large-scale control of vector populations. The aim of the study was to evaluate this new approach using pyriproxyfen on the riverine species *Glossina palpalis gambiensis* (Vanderplank, 1949) in the laboratory. The contamination dose and persistence of pyriproxyfen on sterile males, the impact of pyriproxyfen on male survival, and the dynamics of pyriproxyfen transfer from a sterile male to a female during mating, as well as the impact of pyriproxyfen on pupal production and adult emergence, were evaluated in the laboratory. For this purpose, a method of treatment by impregnating sterile males with a powder containing 40% pyriproxyfen has been developed. The results showed that the pyriproxyfen has no impact on the survival of sterile males. Pyriproxyfen persisted on sterile males for up to 10 days at a dose of 100 ng per fly. In addition, the horizontal transfer of pyriproxyfen from a treated sterile male to a female during mating could be measured with an average of 50 ng of pyriproxyfen transferred. After contacts without mating, the average quantity transferred was more than 10 ng. Finally, the pyriproxyfen powder was very effective on *G. p. gambiensis* leading to 0% emergence of the pupae produced by contaminated females. These promising results must be confirmed in the field. A large-scale assessment of this boosted pyriproxyfen-based SIT approach will be carried out against tsetse flies in Senegal (West Africa).

## Introduction

On the African continent, tsetse flies (Diptera: *Glossinidae*) are the main vectors of the parasites responsible for Human African Trypanosomosis (HAT), or sleeping sickness, and Animal African Trypanosomosis (AAT), also called Nagana^1^. These diseases caused by Trypanosomatidae belong to the group of neglected tropical diseases (NTDs), occurring in developing areas^2^. Sub-Saharan African countries suffer from a significant impact of NTDs on public health and economic development^3^. *Glossina palpalis gambiensis* (Vanderplank, 1949) is one of the main vectors of the HAT parasite in West Africa and is responsible for the persistence of many residual outbreaks of this disease in forest and savannah areas^4^. There is still no vaccine against HAT and curative treatments are difficult to access for the most exposed and vulnerable populations^5^. *Glossina palpalis gambiensis* is also involved in the transmission of AAT, which affects livestock and reduces animal production thus limiting the availability of food resources^6,7^. Despite the millions of doses of trypanocides administered, nearly 3 million cattle die each year in Africa from AAT. Annual direct and indirect agricultural losses attributed to this disease are estimated at more than US$ 4 billion^8^. In addition, many wild bovids infected with trypanosomes do not suffer from serious clinical disease and are thus an important reservoir of infection. The presence of tsetse flies in Africa limits access to millions of square kilometres of fertile and resource-rich land^7^.

To limit the impact of trypanosomoses in Africa, it is necessary to interrupt the trypanosome transmission cycle by reducing the host-vector contact by controlling tsetse fly populations. This control can be achieved through a variety of techniques^9^, including traps, insecticide impregnated screens^10^, live bait technique^11,12^, sequential aerosol technique^13^, and the sterile insect technique^14^ (SIT).

SIT is based on the mass release of irradiated sterile males. This genetic control technique has shown interesting results in controlling tsetse flies^15^. Irradiation causes a multitude of random dominant lethal mutations, leading to infertility^16^. Sterilized males compete for mating with fertile wild females, reducing population fecundity and causing population collapse^17^. SIT proved effective in 1997 on the Island of Unguja (Tanzania) where it eradicated the local vector of AAT, *Glossina austeni* (Newstead, 1912), allowing livestock development^14–18^. SIT is most effective when the wild population is isolated and its density has already been reduced by trapping or using insecticide-impregnated screens or sequential aerial technique^15^. Nevertheless, the release of sterile insects is effective provided that sterile males have the ability to fly, survive and compete with their wild counterparts and that sufficient ratio of sterile to wild males can be achieved^19,20^. The successful mating between sterile males and wild fertile females is mandatory for a successful SIT program. It is estimated that an average ratio of 10 sterile males released to one wild male would compensate for the lower competitiveness of sterile males compared to wild males in the field^21^.

However, SIT faces many technical and logistical constraints, particularly when it comes to treating tsetse populations over large areas such as in continental Africa^21^. This is why a new control method called “boosted SIT” has been proposed. It combines SIT and the self-dissemination of a biocide by the insect^22^. The principle of self-dissemination of a biocide is to allow the horizontal transfer of this biocide between two insects of the same species, for example from the male to the female through their mating^23^. The hypothesis is that a simple contact between sterile males and wild females is enough to disseminate the biocide even if the mating attempts fails.

Here, the biocide is a juvenile hormone analogue, pyriproxyfen (PP), which acts by mimicking the action of the juvenile insect hormone^24^. Like other insect growth regulators (IGRs), PP is toxic to a broad spectrum of insects during their developmental stages. Pyriproxyfen has traditionally been used in aquatic habitats to prevent mosquito larvae and pupae from developing into adults^25^. In addition, PP also has an impact on fecundity and fertility of adult mosquito females through tarsal contact. Pyriproxyfen affects both the production and development of eggs and adult emergence^26,27^. Studies on *Aedes* mosquitoes have showed that exposure to PP can reduce the reproductive capacity of mosquito females^28–30^. Moreover, a study conducted with *A. aegypti* has shown that PP could be transferred from treated males to virgin females during mating, and then subsequently transferred from females to water-holding containers at concentrations that inhibited emergence^31^. For tsetse flies, adult females of *Glossina morsitans morsitans* (Wiedemann, 1850), exposed to a dose as low as 0.01 µg of PP produced non-viable offspring for at least two reproductive cycles^32^. The same study showed that males of *G. m. morsitans* exposed to 0.1 µg of PP could transfer a sufficient dose during mating females to cause sterility in their mates.

Pyriproxyfen has no mutagenic or toxic effect on mammals at the doses recommended by WHO since it targets an insect-specific metabolic pathway^33^. It is used against pests of public health interest (houseflies, cockroaches), agricultural pests (aphids, whiteflies) and pathogen-carrying insects^24^ (mosquitoes) for which it is authorized in drinking water^33^. In addition, no resistance of tsetse flies to this biocide is currently known. The Boosted SIT method applied to tsetse consists of the mass release of male tsetse flies sterilized by radiation and coated with biopesticides, in this case PP, into wild tsetse fly populations. Thus, males will sterilize not only wild virgin females, but also already inseminated females. Previously inseminated females generally refuse to mate again, but may still receive a biocide during mating attempts of treated sterile males. Indeed, sexual harassment of females, even those already inseminated, by males is common among tsetse and even causes increased mortality^34^.

The technique of contaminating sterile males with PP is an important part of this new approach. We assume that PP is more easily transferred to the female during mating when it is located on the outer part of the male’s body. Once on the female, part of the PP can be transferred to the larva *in utero* affecting its development as shown previously^32^. In addition, it is possible that a quantity of PP, present on the female, may be deposited on the larva during delivery. Thus, the biocide could have an impact on pupal development and adult emergence^35^.

This innovative method could be used to treat sterile tsetse males before their aerial release by aircraft or by drones^36^. The ultimate goal of the project is to eliminate targeted populations of riverine tsetse flies in at risk areas of tropical Africa, after conducting epidemiological and socio-economic studies on these populations.

The goal of our study was to evaluate in *G. p. gambiensis*, the impact of a solid PP formulation on sterile male survival, its persistence on treated males and its transfer rate to females during mating as well as its impact on their progeny production.

## Results

### Sterile treated males’ survival

**Figure 1:**
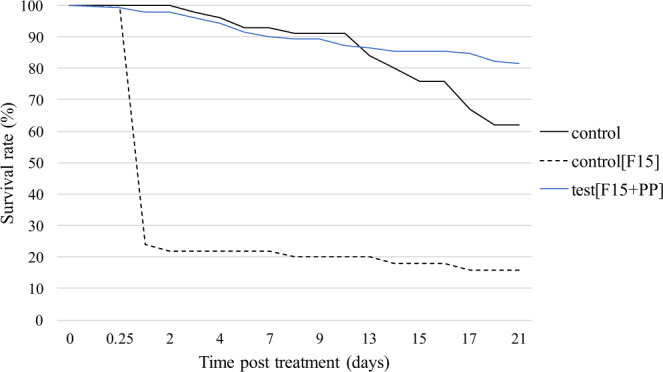
Survival rate of sterile males of *Glossina palpalis gambiensis* over 21 days. The control group is composed of *n* = 50 untreated flies and the control [F15] group of *n* = 50 flies treated with powder F15. The test [F15 + PP] represents the average survival rate of five groups each containing *n* = 50 flies treated with F15 powder containing 40% pyriproxyfen.

Survival rate of PP-treated sterile males remained stable for the first three days of the experiment, then decreased until 21 days’ post treatment, staying above 75% (Fig. 1). The control flies’ survival rate (black line) was similar to the average survival rate of PP-treated groups (blue line) for the first 10 days, then fell below 65% until day 21 of the experiment. No significant difference in survival rate was noted between treated and control groups (Cox test: p = 0.55). Unexpectedly, the survival rate of the control treated with the F15 carrier powder only (formulation without PP) (black dash line) dropped rapidly to less than 25% only 24 hours after treatment.

### Pyriproxyfen persistence

**Figure 2:**
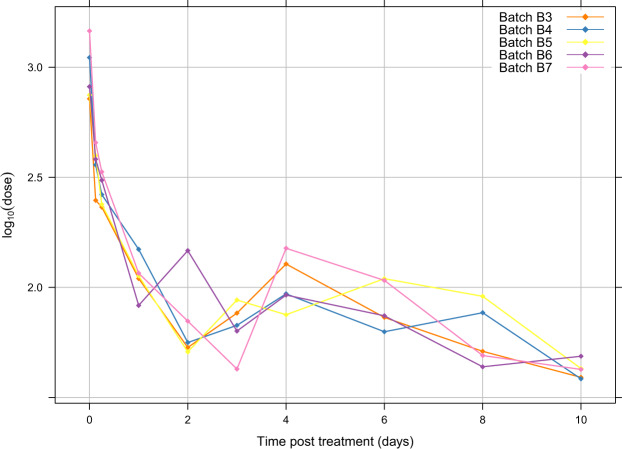
Persistence of pyriproxyfen (log scale) over time after treatment of sterile males of *Glossina palpalis gambiensis* with F15 + PP (*n* = 2 flies/time/batch).

The amount of PP on treated sterile males decreased over time after treatment (Fig. 2). There were some variations in PP amount between each batch of treated sterile males. The average PP amount per sterile male fell from about 1,000 ng just after treatment to below half that amount in only three hours. After 24 hours, the average PP dose stabilized around 100 ng until ten days. The average loss of PP by sterile males was 63% three hours post-treatment and 88% after 24 hours.

### Pyriproxyfen transfer from treated sterile male to female during mating

All the 60 virgin females used for this experiment mated with the sterile males treated with PP. The average amounts of PP present on sterile males after mating, three and 24 hours after treatment, were 365.5 ± 133.8 ng and 218.1 ± 73.6 ng respectively (Table 1). This difference was significant (Wilcoxon test: p < 0.001).

**Table 1:** Mean (±se) amounts (ng) of pyriproxyfen traced on females, mated sterile males and unmated sterile males at three or 24 hours after male treatment.

Treatment	Number of flies	Time after male treatment (h)	
		3	24
Sterile male unmated	10	859.9 ± 70.4	500.1 ± 120.0
Sterile male mated	30	365.5 ± 24.4	218.1 ± 13.4
Female	30	49.2 ± 2.1	50.2 ± 1.2

Females mated with males treated three hours or 24 hours before mating received an average of 49.2 ± 11.7 ng and 50.2 ± 6.7 ng of PP respectively (Table 1), which was not significantly different (Wilcoxon test: p = 0.3285). In addition, there was no relationship between the duration of mating (from 20 minutes to 2 hours) and the amount of PP received by the female (data not shown).

### Pyriproxyfen transfer from treated sterile male to female during contacts without mating

**Figure 3:**
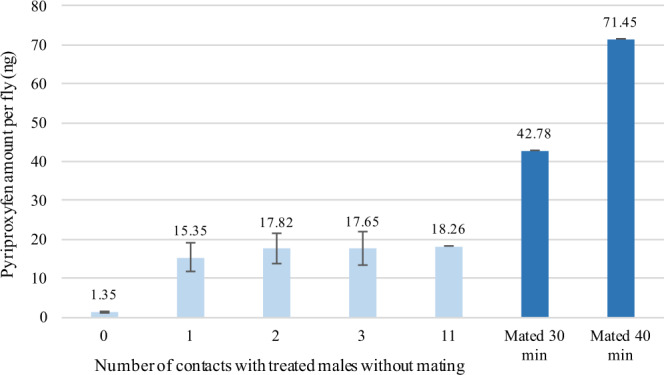
Mean (±se) quantity of pyriproxyfen per female already inseminated according to the number of contacts (without mating) with treated male; only two females accepted to mate, 30 and 40 minutes, with treated male (right of the graph).

Of the 30 couples tested, only two females already inseminated by fertile males accepted a second mating with a treated sterile male. One female mated for 30 minutes and received 42.78 ng of PP and a second female for 40 minutes and received 71.45 ng of PP (Fig. 3). An average amount of 1.35 ± 0.54 ng PP was found for four females that had no contact with a treated sterile male. This minimal amount represents the background noise for quantification of PP using HPLC. Eleven females had a single contact, nine females had two contacts, three females had three contacts and one female had 11 contacts, resulting in an average of 15.35 ± 12.49 ng, 17.82 ± 11.68 ng, 17.65 ± 7.16 ng and 18.26 ng of PP per female respectively (Fig. 3). According to the non-parametric Kruskal-Wallis test, the “number of contacts” factor had a significant positive effect on the amount of PP present per female already inseminated (p = 0.032), even though the increase in dose was small.

### Pyriproxyfen impact on pupal production and adult emergence

#### Female survival

The survival rates of females in all groups did not fall below 50% after 35 days stored in a climatic chamber at the insectarium (See Supplementary Fig. S1). The shape of the survival curves was similar for all groups except for control group C1 (females mated with fertile untreated males, located in a separate insectarium from the other groups) and control group C2 (females mated with fertile untreated males). The survival rate of these two control groups dropped more rapidly at the beginning of the experiment than that of the other groups reaching 59% in group C1 and 55% in group C2 at the end of the experiment. There was a significant difference between the survival distribution of group C4 (females mated with sterile untreated males) and group C1 (Log-rank test: p = 0.00463). Moreover, there was a significant difference between the survival distribution of group C2 and that of group C4 (Log-rank test: p = 0.00185).

#### Pupal production and adult emergence

Table 2 shows the results of this experiment and the characteristics of each group used. There are differences in number of pupae per initial female and emergence rate in all groups during the 35-days follow-up. Group C2 is a control group considered to be 100% fertile because it is composed of females mated with untreated fertile males. In the control group C2, 1.2 pupae per initial female were produced, whereas in group C4, 0.02 pupae per initial female were produced resulting in a 98% reduction in pupal production. Group T2 females produced 0.05 pupae per initial female corresponding to a 96% reduction compared to the control group C2. In group T7, 0.02 pupae per initial female were produced, a reduction of 98% compared to the control group C2. In groups T3 and C5, 0.35 and 0.85 pupae per initial female were produced respectively, resulting in a reduction in pupal production of 71% in group T3 and 29% in group C5. In all the other groups there was no significant reduction (NR).

**Table 2:** Pupal production and adult emergence of *Glossina palpalis gambiensis* resulting from the females mated as different cross schedules treated with the fertile, PP, sterile males and their combinations.

Treatment	Cross schedule	Initial females (No.)	Pupal Production (No.)	Abortions (No.)	% Pupal Reduction	% Adult Emergence
Control groups	**C1** ♀ + fertile ♂ (external laboratory)	44	50	—	NR (1.14)*	96
	**C2** ♀ + fertile ♂ (climate chamber A)	40	48	9	NR 1.20	0
	**C3** ♀ + fertile ♂ **(climate chamber B)**	40	85	5	NR(2.13)	0
	**C4** ♀ + sterile ♂	43	1	52	98(0.02)	0
	**C5**♀ +[50% fertile ♂ - 50% sterile ♂]	40	34	26	29(0.85)	0
Treated groups	**T1** ♀ + PP fertile ♂	45	80	18	NR (1.78)	0
	**T2** ♀ + PP sterile ♂	42	2	64	96(0.05)	0
	**T3** ♀ +[50% fertile ♂- 50% PP sterile ♂]	34	12	26	71(0.35)	0
Treated groups	**T4** ♀ + fertile ♂ replaced by sterile ♂ after 24 h	40	63	12	NR(1.58)	0
	**T5** ♀ + fertile ♂ replaced by PP fertile ♂ after 24 h	40	68	9	NR(1.70)	0
	**T6** ♀ + fertile ♂ replaced by PP sterile ♂ after 24 h	40	69	6	NR(1.73)	0
	**T7** ♀ + sterile ♂ replaced by PP sterile ♂ after 24 h	45	1	60	98(0.02)	0

*Parenthesis indicates the pupae/initial female; NR- means no reduction.

In addition, the number of aborted eggs per initial female in the control group C2 was 0.23 while in groups C4 and T2 it was 1.21 and 1.52 respectively. In group T7, 1.33 aborted eggs were produced per initial female while groups T3 and C5 showed 0.76 and 0.65 aborted eggs produced per initial female, respectively.

**Figure 4:**
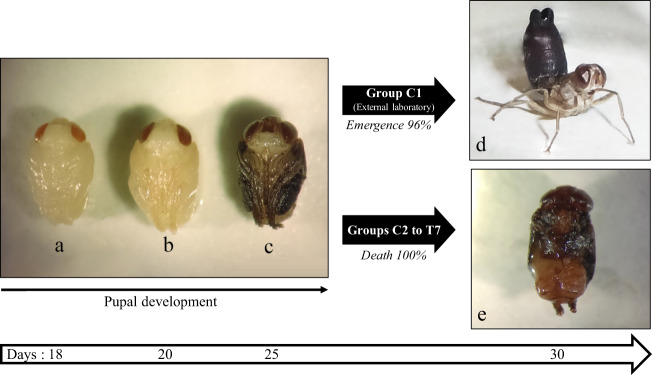
Development stages of pupae during the experiment on the effect of pyriproxyfen on adult emergence of *Glossina palpalis gambiensis* after dissection from their puparium. (**a**–**c**) pupae after 18, 20 and 25 days of development respectively (© IRD - Lison LAROCHE). (**d**) a viable emergent fly (© CIRAD – Bernadette TCHICAYA). (**e**) a fly that died in its puparium (© IRD - Lison LAROCHE).

All dissected pupae were similar in all groups C1 to C5 and T1 to T7 (i.e. with or without males previously impregnated with PP) at each stage of the pupal development, from 18 to 25 days (Fig. 4). Pupae from group C1 couldn’t be dissected because they all emerged. This is why we dissected the control pupae stored in the same conditions and same insectarium as the group C1 pupae. All pupae grew correctly: wings, proboscis, arista and small hairs were observed during development and turned grey (Fig. 4a–c). Only in group C1, located in an insectarium isolated from the other groups (5 km), did adults emerge and gave viable flies (Fig. 4d, 48 adults from 50 pupae) while in groups C2 to C5 and T1 to T7, no adults emerged. In groups C2 to C5 and T1 to T7, we observed body movements of dissected pupae only up to day-25 of pupation, a sign of life in the flies; whereas after 30 days (expected emergence date) only brown flies, with brittle tissue and a bad smell of putrefaction were observed, a characteristic sign of death (Fig. 4e).

## Discussion

This work demonstrated that our powder formulation containing 40% PP did not affect the survival of sterile males of *G. p. gambiensis* over three weeks. Previous studies have shown that PP impairs neither adult insect activity nor life expectancy^37^. This result is of great importance with regard to their release in the field in the framework of an SIT program. Unexpectedly, PP-free powder F15 caused a high mortality rate in tsetse flies (Fig. 1). Pyriproxyfen has a higher density than the other components used in F15 powder. As a result, the F15 powder has a lower density than the F15 + PP powder. For the same mass as the powder F15 + PP, the volume applied of the F15 powder was greater and could have blocked the spiracles of tsetse flies and cause their death by asphyxiation. In addition, this density difference between the powders could lead to slightly different binding properties on tsetse flies. The F15 powder has a greater negative electrostatic charge due to the absence of PP and makes its attachment to the tsetse stronger and more difficult to remove by the insect during grooming.

Although we observed a rapid decrease in the surface amount of PP in treated sterile males, an average amount of 100 ng PP per specimen persisted from 24 hours to 10 days post-treatment on the surface of treated males. This amount found on males is enough to cause disruption of the reproductive potential of its mate, by assuming a transfer rate from males to females of 10%, as demonstrated by previous work on *G. m. morsitans*, a savannah species of similar size to our riverine species^38^. Since sterile males released in the field have a mean lifespan of 10 days or less^19^, the persistence of 100 ng PP on the surface of treated sterile males for 10 days is very promising from an operational point of view.

The rapid decrease in the amount of PP on the surface of treated sterile males (Fig. 2) could result from (i) the elimination of the F15 + PP formulation by fly grooming activities (usually observed), (ii) shedding off of the powder itself from the cuticle of treated flies, (iii) penetration of the cuticle of a part of the PP, as shown in *Hyposoter didymator* (Thunberg, 1822)^39^. In addition, the persistence of the PP molecule itself, without interaction with the tsetse fly, varies according to ambient conditions (temperature, humidity, pH, ultra violet (UV) radiation, etc.) and the environment in which it is found^40^.

To determine the amount of PP transferred from a treated sterile male to a female, individual mating was performed at three and 24 hours after treatment of the sterile males. Three hours represents the time of processing, conditioning and transport of sterile males to release points as part of an operational aerial release SIT program against tsetse^19^, so that mating in the field could not take place before.

With the F15 + PP powder formulation, females received an average amount of 50 ng PP after mating with coated sterile males when mating occurred three or 24 hours after treatment (Table 1). This average dose was greater than 10 ng PP, an amount that can disrupt the reproductive potential of another tsetse fly species^38^. Thus, even if sterile males treated with F15 + PP, when released for an operational campaign of “Boosted SIT”, take up to 24 hours to find and mate with a female, the dose of PP transferred to the female would be enough to have an impact on its reproductive potential.

In previous work, a liquid formulation containing 20% of PP (F8) was used on *G. p. gambiensis*^41^. The amount of PP found on the surface of males after treatment with F8 was much lower than when using the powder formulation F15. This can be explained by a poorer attachment of the PP on the male with the liquid formulation F8 than with the powder formulation F15. With F8, a high heterogeneity in the amount of PP on the surface of treated males was observed, whereas with F15, amounts were more homogeneous. The persistence of PP was lower on the males treated with F8 than with the F15, probably because the liquid formulation F8 was directly absorbed within the body of the male instead of remaining at the surface. However, we did not measure the internal concentration to confirm this hypothesis. In addition, the amount of PP transferred to females by F8-treated males was too small to expect a significant impact on adult emergence^38^. This PP uptake was reported to be around 99% three days after treatment in another insect model, the parasitoid *Hyposoter didymator*^39^.

According to previous work if the PP transfer rate approaches 10%, which corresponds to 10 ng of PP transferred from the male to the female, this quantity would be sufficient to completely sterilize the female^38^. This is why we decided to switch from the liquid formulation of PP to the F15 powder formulation that could better fix a higher quantity of PP on the surface of the male body^41^. The higher amount of PP transferred to females with the F15 + PP powder formulation could be explained by a higher initial dose of PP (40%), better adhesion (due to the properties of the formulation) and a higher powder concentration of PP in areas of treated males more conducive to transfer. Since the cuticle of insects is positively charged, it attracts the negative charges present in the F15 + PP formulation. Moreover, if PP powder is present on the claspers of the male, powder particles may enter the reproductive tract of the female during mating. We also observed that males tend, a few minutes before the end of mating, to make circular movements with their hind legs on their partner’s abdomen. These movements are likely to occur during transport of the spermatophore from the male reproductive system to the uterus of the female^42^. They could, by their frequency and duration, facilitate the transfer of PP to the female. In addition, an active molecule in its solid form (crystals) is generally less sensitive to environmental conditions than in its liquid form or dispersed in a solvent medium (water or organic solvent)^43^. At room temperature, PP is a solid (melting temperature is 47 °C). This form is very stable, allowing it to be stored longer than in liquid form^24^. Thus, the choice and development of a new formulation of PP in powder form has proved to be judicious and these transfer results obtained using with the F15 + PP powder suggest a good biological efficacy. We have also shown that a transfer of PP occurred even after a single contact between male and female without mating. This is of great interest for the Boosted SIT application: in addition to targeting virgin females, treated sterile males may also, through their harassing behaviour, attempt to mate with females that have already been inseminated by wild males. However, PP acts after fertilization, during the pupation stage^25^. Generally, females refused to mate when they had already mated once. However, we have shown here that two out of 30 females already inseminated accepted a second mating. This behaviour of re-mating is consistent with observations made in wild populations of *G. fuscipes fuscipes* (Newstead, 1910)^44^. In addition, we showed that all of the 24 females already inseminated that refused to mate but had at least one contact without mating received PP (Fig. 3). A single contact without mating was sufficient for the female to receive more than 10 ng of PP. This result is interesting for the application of the Boosted SIT, due to the very frequent sexual harassment behaviours by male tsetse flies. This should be confirmed under semi-experimental conditions, or even in the field.

To determine the impact of PP on female survival, a survival study was conducted during the experiment. We demonstrated that PP did not affect the survival of females mated with treated males over 35 days. Differences in the distribution of survival rates were observed between females mated to fertile males (control groups C1 and C2) and those mated to sterile males (group C4). Indeed, sterilized females tend to have a higher survival rate than fertile females because they use less energy during their lifetime to produce offspring than fertile females.

There is a significant reduction in the pupal production in groups C4, T2 and T7 (98%, 96% and 98% respectively) because females mated with sterile irradiated males only (Table 2). The irradiation of males has shown its effectiveness in our experiment. We have also noticed the presence of a large number of aborted eggs in groups of females mated with sterile males, which is consistent with the fact that females mated with sterilized males tend to expel the dead embryo soon after embryonic development stops^45^. There was no significant reduction in pupal production in the two groups where fertile males were replaced 24 hours later by PP-treated or non-sterile males (groups T4 and T6) even though the already mated females accepted a second mating 24 hours later. It has been described that many females of *G. f. fuscipes* keep sperm from different partners, which could be used for insemination. Thus, the storage of sperm from more than one male generates the possibility of sperm competition for fertilization^44^.

In our experiment with *G. p. gambiensis*, the high production of pupae by females may indicate that it was the sperm of fertile males, transferred during the first mating, that was used for fertilization, probably due to sperm precedence^46^. There was no reduction in the number of pupae produced when females were mated with fertile males treated with PP, indicating that PP did not affect the pupal production of *G. p. gambiensis* females.

After about 30 days, there was no emergence from the pupae collected from females in groups C2 to C5 and T1 to T7 while the pupae located in a separate insectarium 5kms away (group C1) produced viable tsetse flies with an emergence rate of 96%, similar to that generally observed in the colonies of this species^47^. As the pupae of the untreated control groups (located in a different climatic chamber from the PP treated groups) did not emerge, it can be assumed that PP contamination has occurred in all flies during the adult handling process. Although all possible precautions had been taken, the F15 + PP formulation is so potent that it has probably spread to all tsetse flies and has resulted in 100% pupal mortality in both replicates. Group C1 was spared this contamination because it was not handled in the same insectarium. Based on these results, we believe that PP principally acts in tsetse flies by disrupting pupal development. The pupal dissections performed at the end of the experiment confirm this hypothesis. Flies exposed to PP develop correctly in the puparium (pupal envelope) but die a few days before their expected emergence (Fig. 4).

Several hypotheses could be made about the mode of action of the PP on the adult emergence of *G. p. gambiensis*. Since the tsetse fly exits the puparium through rhythmic contractions of the ptilinum, a vesicular structure located in front of the fly’s head^48^, it is possible that a sublethal dose of PP irreversibly suppressed the contraction cycles of the ptilinum, causing the fly not to emerge. On the other hand, PP can inhibit the development of the fly’s muscles, causing muscle atrophy preventing the fly from leaving the puparium.

During this laboratory evaluation of the effects of PP on *G. p. gambiensis*, we showed that PP does not have a significant impact on the survival of sterile males. Besides, there is enough PP on males 24 hours after impregnation so that they can still transmit a theoretically sufficient dose of PP to a female (during mating) and thus have an impact on adult emergence. In addition, we have shown that horizontal transfer of PP occurs from a sterile male to a female during mating, but also during contact due to failed mating attempts. The new powder formulation (F15) proved to be more effective in terms of PP transfer than the liquid formulation previously used (F8).

This powder formulation of PP has a huge negative impact on adult emergence rate of *G. p. gambiensis*. Moreover, it is necessary to take great care when using the F15 + PP formulation because it is very contaminating under insectarium conditions. In operational programs, it will be necessary to contaminate the sterile males in release centres isolated from the factories producing the sterile males. In view of these results, the Boosted SIT approach, implemented on a large scale, could target simultaneously virgin females (SIT action) and already inseminated females (PP action).

For the Boosted SIT to have an impact on local vector populations, it is also necessary to test, under experimental and field conditions, the competitiveness of sterile males treated with PP compared to wild males, particularly in terms of flight ability and reproductive behaviour. This would allow us to determine the proportion of treated sterile males to be released in relation to the wild male population for this method to be effective. The ratio of “sterile male treated with PP: wild male” also depends on population size and dynamics as well as the climatic and environmental context. In *Aedes* mosquitoes, models predicted that boosting the sterile males with PP might allow the number of sterile males to be released to be reduced by more than 95% to control Dengue epidemics^49^.

The use of sterile males as specific conveyors to infect females with PP, combined with sexual competitiveness with wild males, is an integrated strategy in itself^22^. In the event that the tsetse fly population develops resistance to PP, the transfer of sterile sperm would prevent the spread of resistance. In addition, the effectiveness of SIT increases with population reduction, due to the increase in the ratio of sterile males to wild males, which pushes the population towards elimination. Another possible application of this principle is the specific contamination of wild females by micro-organisms known to control vectors, such as densoviruses, fungi or entomopathogenic bacteria^50–52^.

## Methods

### Tsetse flies

The *G. p. gambiensis* strain used here originated from Burkina Faso and was reared at the International Atomic Energy Agency (IAEA) insectary in Seibersdorf, Austria. Fertile females, and fertile and sterile males were used. Sterile males were irradiated at the pupal stage with 120 gray (Gy) decreasing pupal production by more than 95%^53^. Sterile pupae were transported to the insectarium in Montpellier under chilled conditions (at 10 °C, see^54^ for more details) with Fedex® transport over 24 or 48 hours. Fertile pupae were transported under ambient conditions to the insectarium with the same type and duration of transport. The newly emerged flies were separated by sex manually and put in Roubaud cages (13 × 8 × 5 cm) in groups of 50 adults. The flies were maintained in a climate chamber (Binder KBF240) at 25 ± 1 °C and 80 ± 5% r.h., with a cycle of 12 L:12D.

Three times a week (Monday, Wednesday and Friday), flies were offered a preheated blood meal on an *in vitro* silicon membrane system using defibrinated sheep blood collected aseptically and previously frozen at −20 °C. The feeding system was installed in a climate chamber that was maintained at 25 ± 1 °C and 50 ± 5% r.h. and the system was used to feed flies from all treatments. For optimal feeding of tsetse, the blood temperature was adjusted to approximately 37.2 °C. In order to prevent cross-contamination between batches of treated flies, separate feeding membranes were used for each of the three different tsetse fly groups: (i) controls (ii) treated with F15 powder and (iii) treated with F15 + PP powder (see details below). For each feeding phase, tsetse flies were fed for ten minutes under low light conditions, i.e. in a room with the light off and the door ajar, which promotes blood feeding.

### Treatment with pyriproxyfen

**Figure 5:**
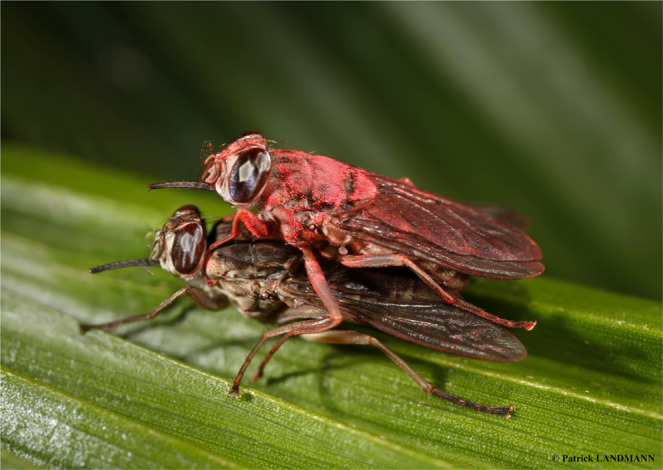
Mating between an untreated female *Glossina palpalis gambiensis* and a male treated with a pyriproxyfen pink powder (© IRD – Patrick LANDMANN).

The males were treated with two types of powder. A PP-free powder called F15 (developed by 3 F Innovation, France, patent pending) was used to treat the control group. The F15 powder has the following characteristics: it attaches easily to the body of the treated insect, it transfers very well from one insect to another during their contact and it incorporates a fluorescent dye that allows its distribution on treated insects to be observed under UV light (Fig. 5). The same powder containing 40% active ingredient of PP (F15 + PP, also developed by 3 F Innovation, France) was used to treat the treatment groups. The PP technical (97% in purity) was obtained from Tagros Chemical India Ltd. The composition of the two powders is similar, with the exception of the biocide in F15 + PP and a small difference in density and particle size due to the presence of PP. To treat the males, in a chemical hood, 35 mg of F15 or F15 + PP formulations were placed in a 140 mL plastic container (height: 72 mm, diameter: 51 mm), previously refrigerated at 7 °C for 20–30 min. The container was then shaken until the powder was evenly distributed over the walls. Fifty caged tsetse males were knocked-down on ice for three minutes and placed in the container. The container was then rolled manually on ice, rotating in one direction and then in the other direction for 25 seconds. Afterwards, the homogeneity of the tsetse fly contamination and between tsetse flies was verified by observing the fluorescence with a binocular microscope using a UV light.

### Quantification of pyriproxyfen

Quantification of the PP deposited on the flies was carried out by high performance liquid chromatography (HPLC) by an analytical chemist at Montpellier University (See Supplementary Method S2 online). Tsetse flies or pupae were placed individually in 4 mL glass flasks containing 1 mL of hexane-fenoxycarb solution (C = 100 ng/mL) and flasks were shaken manually every 15 minutes at room temperature for one hour to extract PP prior to the quantification. Tsetse flies and pupae not treated with PP were also quantified for PP as background noise control. The background value is between 0.1 and 1 ng.

### Survival of pyriproxyfen treated sterile males and pyriproxyfen persistence

Batches of 50 sterile male *G. p. gambiensis* were used. The first control group was not treated but was submitted to the same handling process conducted for the other groups. Flies from the second group were treated with F15. Finally, the five other test replicate groups were treated with F15 + PP. After treatment, all groups of sterile males were stored in the climatic chamber until the end of the experiment. Mortality was recorded daily (except on weekends) for 21 days and used to calculate the survival rate. To assess the persistence of the biocide (PP) on the sterile males, two tsetse flies were removed from each of the five treated groups at different times after treatment (after 30 minutes; 3 and 6 hours; 1, 3, 4, 6, 8 and 10 days) to quantify the PP with HPLC as above.

### Pyriproxyfen transfer between male and female

To demonstrate the horizontal transfer of PP between treated sterile males and females, a preliminary experiment was conducted. Mating was performed three and 24 hours after PP treatment of the sterile males in a room under optimal temperature and humidity conditions.

At three or 24 hours after treatment, the control group of 15 untreated sterile males and both the treated groups of 15 treated sterile males each were mated in pairs with females. For each pair, a 6–7 days old male was placed individually with a 3–4 days old female in a small cardboard cup (180 mL, height: 80 mm, diameter: 74 mm) to increase the frequency of contact. Each cup was closed with a mosquito net that allowed light to enter. The duration of mating was measured by observation for each couple. At the end of the mating, the male and female were removed for individual quantification of PP present on their bodies. In addition, the quantification of PP was performed individually on 10 non-mated sterile males (control) three and 24 hours after treatment with PP.

A second experiment was performed to explore the possibility of the transfer of PP following contacts without mating of a treated male with a female. A group of 50 sterile males (6–7 days old) was treated with PP three hours before mating individually with already inseminated females. The behaviour of each pair was observed and the contacts without mating were counted for one hour. After one hour, males and females were removed for individual quantification of PP present on their bodies, with the exception of non-separated male/female pairs. For mated pairs, the duration of mating was measured and the male and female were removed once separated for individual quantification of PP.

### Pyriproxyfen impact on pupal production and adult emergence

Females 3–5 days old were mated either with fertile or sterile males 7–10 days old, treated or not with PP (Table 2). All groups consisting of females mated with untreated males were placed in a separate climate chamber from groups consisting of females mated with PP-treated males.

Quantification of PP on both untreated and treated males was performed prior to mating in order to control treatment. Mating took place in 27 × 17 × 17 cm net cages, with a male to female ratio of 1:1 to ensure the mating of as many females as possible. Mating cages were placed in the climatic chamber at 25 ± 1 °C and 80 ± 5% r.h., with a 12 L:12D cycle.

We used three control groups C1, C2 and C3, composed of untreated fertile males: C1 was placed in an insectarium (in which there was never PP) located 5 km away from the other groups, C2 was placed in climatic chamber A (containing only untreated males) and C3 was placed in another climatic chamber B (containing only PP-treated males). Another control group C4 used untreated sterile males to confirm the effect of SIT (expected inhibition of approximately 95% of pupal production^53^). The last control group, C5, contained half untreated fertile males and half untreated sterile males (to study the competitiveness between fertile and sterile males).

To examine the effect of PP on pupal production and adult emergence, several experimental groups were set up: T1 using PP treated fertile males (to examine the effect of PP on adult emergence); T2 using treated sterile males (to examine the boosted SIT effect on adult emergence); and T3 using half untreated fertile males and half treated sterile males (to examine the competitiveness of the boosted SIT).

Four experimental groups consisted of first mating females with one type of male for 24 hours, then removing and replacing the used males with another type of male: T4 consisted of untreated fertile males replaced by untreated sterile males (to determine the SIT effect after 24 hours), T5 consisted of untreated fertile males replaced by treated fertile males (to measure the boosted SIT effect by non-mating contacts), T6 consisted of untreated fertile males replaced by treated sterile males (to determine the boosted SIT effect after 24 hours by non-mating contacts), T7 consisted of untreated sterile males replaced by treated sterile males (to measure the SIT effect and then the Boosted SIT effect).

Males in groups T4 to T7 were removed from the mating cages 24 hours after mating began to limit female mortality due to male harassment^34^. Then, the females were transferred to Roubaud cages (13×8×5 cm) and the survival of the females was monitored daily (except on weekends). The mean number of pupae produced per initial female and the rate of adult emergence from these pupae were measured. Female cages were placed on individual larviposition cups. Pupal production was recorded daily by cage (except at weekends) and sorted into normal and aborted eggs. The pupae were then stored by group in Borel® glass jars in the climate chamber until they emerge. Pupae dissections were performed at 18, 20, 25 and 30 days after pupation to monitor internal development. The images of these dissections were taken with a binocular microscope at x10 magnification. Adult emergence and sex sorting were assessed at the beginning of emergence (on average 30 days after the first larviposition). Two replicates of this experiment were conducted. Based on the results of the first (See Supplementary Table S3 online), two additional control groups were added in the second replicate (C1 and C3).

### Data analysis

Data analysis were carried out with R^55^ through Rstudio software version 1.1.447 - © 2009–2018 and Microsoft Excel version 15.20 © - 2016. Normality was tested via a Shapiro test and homoscedasticity via a Fisher Snedecor test according to the data to be analysed. Survival analyses were performed with R “survival” package^56^. Survival functions of PP treated and untreated flies were compared with the semi-parametric Cox model test and the non-parametric Log-Rank test. To compare averages of two independent samples that did not follow a normal distribution, the non-parametric Wilcoxon Mann-Whitney test was used^57^. In order to test relationships between two series of observations that did not follow a normal distribution, we calculated Kendall’s “tau” coefficient^58^. To determine only one factor effect on more than two independent samples not following the normal distribution, the non-parametric Kruskal-Wallis test was used^59^.

## Supplementary information

Supplementary Information.
