# Supplementary material for: Boosting the sterile insect technique with pyriproxyfen increases tsetse flies Glossina palpalis gambiensis sterilization in controlled conditions
Source: Sci Rep. 2020 Jun 19;10:9947. doi: 10.1038/s41598-020-66850-9 (PMC7305199; doi:10.1038/s41598-020-66850-9)
Supplement: Supplementary file 1 — Supplementary Information. [file 41598_2020_66850_MOESM1_ESM.docx]

**Manuscript -** Ref. No.:  SREP-19-40285B

**Title**

Boosting the sterile insect technique with pyriproxyfen increases tsetse flies *Glossina palpalis gambiensis* sterilization in controlled conditions

# Authors

# L. Laroche^1,3^*, S. Ravel^1^, T. Baldet^2^, R. Lancelot^2^, F. Chandre^3^, M. Rossignol^3^, V. Le Goff ^3^, M. Duhayon^2^, J-F. Fafet^4^, A.G. Parker^5ϕ^, J. Bouyer^1,2,5^

^1^ Intertryp, IRD, Cirad, Univ Montpellier, France

^2^ ASTRE, Cirad, INRA, Univ Montpellier, Montpellier, France

^3^ MIVEGEC, IRD, CNRS, Univ Montpellier, Montpellier, France

^4^ 3F Innovation, Saint-Amarin, France

^5^ Insect Pest Control Laboratory, Joint FAO/IAEA Division of Nuclear Techniques in Food and Agriculture, IAEA, Vienna, Austria

^ϕ^ Present address: Roppersbergweg 15, 2381 Laab im Walde, Austria

*Correspondence: lisonlaroche@hotmail.fr

**Supplementary Figure S1:** *Glossina palpalis gambiensis* female survival rate over time after mating with males of each group.

C1 to C5 represents the five control groups and T1 to T7 the seven experimental treated groups. For more details on the definition of these groups, refer to the text.

**Supplementary Method S2:** Protocol used to perform HPLC analyses.

1. *LC-MS/MS conditions*

The LC-MS/MS system consisted of a Schimadzu NexeraX2 series (Shimadzu, Japan) binary pump, vacuum degasser, column oven and auto-sampler system connected to a LCMS-8050 (Shimadzu) triple quadrupole MS equipped with an Electrospray Ionization Source (ESI). LabSolutions was used for system control, data processing and acquisition.

Chromatographic separation was performed by a Biphenyl kinetic analytical column (2.6 µm, 100 Å, 50 x 2.1 mm). The column temperature was maintained at 40°C. The temperature of the autosampler was set at 15°C. Eluent A was water with 0.1% formic acid, and Eluent B was acetonitrile with 0.1% formic acid. The flow was 0.5 mL/min. The gradient started at 20% B and went to 100% B in 3 min. Then, the gradient was plateau of a 1 min with 100% B followed by a return to initial conditions. The run time was 7 min.

The MS/MS system was performed under positive ESI and the Multiple Reaction Monitoring (MRM) mode was used to identify compounds of interest. The operational parameters of the MS were as follows; argon as a collision gas, interface voltage at 4 kV, fogging gas flow rate 3L/min, heat gas flow rate 10 L/min, interface temperature 300°C; DL temperature 250°C, heat block temperature 400°C and drying gas flow rate 10 L/min. Detections of the analytes were performed using MRM mode to monitor transitions between precursor and product ions from 322˃96 for pyriproxyfen and 302˃ 88 for IS (Fenoxycarb).

1. *Preparation of stock and standard solutions*

Stock solutions of pyriproxyfen and IS were prepared separately in acetonitrile at a target concentration of 1 mg/mL and aliquoted for storage. Stock working solutions of pyriproxyfen were prepared by diluting the stock solution with acetonitrile (1 – 1000 ng/mL) and quality control (QC) samples (8.80 and 800 ng/mL).

1. *Sample preparation*

The flies are dipped in 1 mL of cyclohexane containing 100 ng/mL of IS. After agitation, the supernatant is removed and evaporated to dryness before being absorbed in 1 mL of acetonitrile. The volume injected is 5 µL.

**Supplementary Table S3:** Pupal production and adult emergence of *Glossina palpalis gambiensis* resulting from the females mated as different cross schedules treated with the fertile, PP, sterile males and their combinations (first replicate).

| Treatment | Cross schedule | Initial  females | Pupal  Production | Abortions  (No.) | % Pupal  Reduction | % Adult  Emergence |
| --- | --- | --- | --- | --- | --- | --- |
|  |  | (No.) | (No.) |  |  |  |
| Control groups | **C1** | - | - | - | - | - |
|  | **C2** | 46 | 109 | 3 | NR | 0 |
|  | **♀** + fertile **♂**  (climate chamber A) |  |  |  | (2.37) |  |
|  | **C3** | - | - | - | - | - |
|  | **C4** | 49 | 3 | 78 | 97 | 0 |
|  | **♀** + sterile **♂** |  |  |  | (0.06) |  |
|  | **C5** | 42 | 52 | 14 | 48 | 0 |
|  | **♀** +[50% fertile **♂** - 50% sterile **♂**] |  |  |  | (1.24) |  |
| Treated groups | **T1**  **♀** + PP fertile **♂** | 46 | 107 | 9 | NR  (2.33) | 0 |
|  |  |  |  |  |  |  |
|  | **T2** | 45 | 3 | 65 | 97 | 0 |
|  | **♀** + PP sterile **♂** |  |  |  | (0.07) |  |
|  | **T3** | 44 | 91 | 11 | 13 | 0 |
|  | ♀ +[50% fertile ♂- 50% PP sterile ♂] |  |  |  | (2.07) |  |
|  | **T4** | 45 | 98 | 5 | NR | 0 |
|  | ♀ + fertile ♂ replaced by sterile ♂ after 24h |  |  |  | (2.18) |  |
|  | **T5** | 46 | 116 | 10 | NR | 0 |
|  | ♀ + fertile ♂ replaced by PP fertile ♂ after 24h |  |  |  | (2.52) |  |
|  | **T6** | 48 | 90 | 11 | 21 | 0 |
|  | ♀ + fertile ♂ replaced by PP sterile ♂ after 24h |  |  |  | (1.88) |  |
|  | **T7** | 39 | 5 | 58 | 95 | 0 |
|  | ♀ + sterile ♂ replaced by PP sterile ♂ after 24h |  |  |  | (0.13) |  |

*parenthesis indicates the pupae/initial female; NR- means no reduction
